# Negative Paper Spray Ionization Mass Spectrometry for the Determination of Endocrine-Disrupting Chemicals with Application to Paraben Analysis in Cosmetics

**DOI:** 10.3390/molecules30224356

**Published:** 2025-11-10

**Authors:** Seonyoung Cho, Sarmila Shrestha Amatya, Hyerin Bahng, Eungyeong Lee, Yunsang Ko, Sangwon Cha

**Affiliations:** Department of Chemistry, Dongguk University, Seoul 04620, Republic of Korea; chosy1229@naver.com (S.C.); amatyaz@gmail.com (S.S.A.);

**Keywords:** paraben, endocrine-disrupting chemicals, cosmetics, paper spray ionization, tandem mass spectrometry

## Abstract

Paper spray ionization mass spectrometry (PSI-MS) enables rapid analysis with minimal sample preparation, yet negative-ion mode performance has been limited by poor sensitivity and unstable signals, similar to conventional electrospray ionization. In this study, we optimized negative PSI tandem MS (MS/MS) for twelve endocrine-disrupting chemicals (EDCs) and related biomarkers—including bisphenols, phthalates, parabens, and substituted phenols—used as model analytes. A systematic solvent and additive screen identified 1 mM ammonium fluoride in methanol and 0.1% ammonium hydroxide in 9:1 MeOH/carbon tetrachloride as optimal conditions, providing enhanced deprotonated-ion intensities and improved stability. Calibration curves generated under these conditions showed excellent linearity, with limits of quantitation (LOQs) in the low-ppb range. Application to cosmetic formulations demonstrated reliable paraben quantitation. In fortified hand cream, LOQs below 1 mg/kg were achieved, with recoveries of 93–110% and intra- and inter-day precision below 10% RSD. Notably, PSI-MS/MS performance was comparable to LC–MS/MS, without a separation step. These results demonstrate the feasibility of optimized negative PSI-MS as a sensitive and robust tool for paraben determination in cosmetics and highlight its potential as a versatile platform for broader EDC quantification.

## 1. Introduction

Paper spray ionization (PSI) is an ambient ionization technique for mass spectrometry (MS) in which a small volume of sample is deposited onto a triangular paper substrate and, after drying, a spray solvent and high voltage are applied to the paper tip to produce a plume of charged droplets [1,2,3]. In this process, ionization proceeds in a manner analogous to conventional electrospray ionization (ESI) [2,3]. PSI-MS has been demonstrated as a rapid and quantitative ambient method for analyzing complex samples (e.g., biofluids) without extensive sample preparation [1,4]. Because PSI relies on an ESI-like mechanism, solvent and additive optimization strategies established for ESI can also guide PSI method development. However, PSI also inherits the well-known challenges of negative-ion mode in ESI.

Negative-ion mode ESI (negative ESI) generally exhibits lower sensitivity and poorer stability than positive-ion mode. Corona discharge at the emitter tip is a major concern: when the applied voltage exceeds a critical threshold, electrons are released into the surrounding gas, resulting in unintended ionization of ambient gases and solvent molecules [5,6]. This phenomenon leads to elevated background noise, unstable signals, and, in severe cases, arcing that not only reduces ion current but may also damage MS components [5,6]. The problem is particularly pronounced with water-rich solvent systems, which are especially prone to corona discharge and yield very low ion currents [7]. In addition, negative sprays tend to generate diffuse plumes due to charge repulsion, thereby reducing ion transmission efficiency into the MS inlet [6,8].

Several approaches have been reported to overcome these limitations. Solvent modification is particularly effective: Cole and co-workers demonstrated that chloroform (CHCl_3_)/methanol (MeOH) mixtures improve stability in negative ESI because halogenated solvents act as efficient electron scavengers [6]. Likewise, trace levels of electron-capturing gases such as O_2_ or SF_6_ can suppress corona discharge at the source [6,9]. More recently, Håkansson and colleagues showed that the addition of 0.2% trifluoroethanol to aqueous sprays significantly stabilized negative ESI, extending spray lifetime and enhancing signal intensity by approximately six-fold [7]. This stabilization has been attributed to the preferential evaporation of trifluoroethanol and its ability to capture free electrons, thereby suppressing corona discharge and enabling more reliable negative-ion generation [7].

In negative ESI, the incorporation of additives that facilitate analyte deprotonation in solution or gas phase has been shown to improve sensitivity and reproducibility. For example, ammonium fluoride (NH_4_F, AmF) markedly enhances negative ESI responses [10,11,12]. In a comparative study, the addition of AmF to the mobile phase improved negative-mode sensitivity by 2–22-fold across a wide range of small molecules, far surpassing conventional additives [12]. Ammonium hydroxide (NH_4_OH, AmOH) and acetic acid have also been reported to improve negative ESI responses for bisphenols [13], lipids [14], and nucleoside antiviral agents [15]. In some cases, small fractions of chlorinated hydrocarbons such as CHCl_3_ and carbon tetrachloride (CCl_4_, CTC) mixed into a polar solvent have been found to stabilize certain anion species and improve signal intensity [6,16,17].

Instrumental advances have also enhanced negative-mode stability. Li et al. developed capillary vibrating sharp-edge spray ionization, where a metal capillary emitter is mounted on a vibrating glass substrate. Operating without nebulizing gas, this platform suppressed corona discharge and yielded 10–100-fold higher ion currents with 3–10-fold improved S/N relative to conventional ESI. [18]. Zhou and Pawliszyn enhanced the coated-blade spray emitter by adding a small insulating barrier at the blade tip, which suppressed discharge currents and reduced signal variability to below 10% RSD in negative-mode ambient spray [8].

In this study, we extended these advances to negative PSI. A panel of 12 endocrine-disrupting chemicals (EDCs) and related biomarkers—including bisphenols, phthalates, parabens, and substituted phenols—was selected as the target analytes. These compounds are environmentally important and are typically measured as deprotonated anions in liquid chromatography–tandem mass spectrometry (LC-MS/MS) analyses [19,20,21,22,23,24]. Furthermore, PSI-MS methods have already been demonstrated for several of these chemicals, motivating their selection as representative analytes [25,26,27,28]. We systematically evaluated a range of spray solvents and additives for their ability to enhance ion yields in negative PSI. The most promising solvent systems, based on signal intensity and stability, were then used to construct calibration curves and determine limits of quantitation (LOQ) in solution. Finally, the optimized negative PSI–MS method was applied to the analysis of parabens in cosmetic formulations, and its quantitative performance was assessed.

## 2. Results and Discussion

### 2.1. Optimization of Negative PSI for EDC-Related Model Analytes

#### 2.1.1. Target Analytes

**Figure 1:**
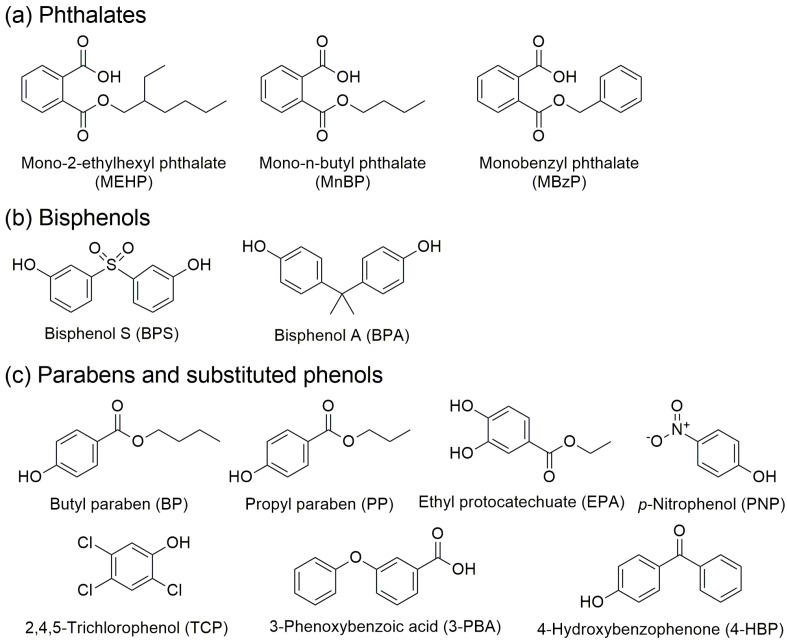
Twelve EDC-related model analytes for negative PSI–MS optimization.

To optimize negative PSI–MS against EDCs and their biomarkers, twelve compounds were selected as model analytes. These compounds are typically analyzed in negative ion mode MS due to the presence of acidic or phenolic functional groups, which readily undergo deprotonation. The twelve analytes were grouped into three categories—bisphenols, phthalate metabolites, and parabens and substituted phenols—and their chemical structures are shown in Figure 1.

Phthalates such as di-(2-ethylhexyl) phthalate, di-n-butyl phthalate, and butyl benzyl phthalate are widely used as plasticizers in consumer products and are well-recognized EDCs. In the human body, they are rapidly hydrolyzed to their corresponding monoester metabolites—mono-2-ethylhexyl phthalate (MEHP), mono-n-butyl phthalate (MnBP), and monobenzyl phthalate (MBzP) (Figure 1a)—which are excreted in urine and serve as robust biomarkers of exposure [29,30]. Bisphenol A (BPA) and bisphenol S (BPS) are widely used as monomers in polycarbonate plastics and epoxy resins (Figure 1b). BPA has been extensively studied and is widely recognized as an EDC with estrogenic activity and frequent urinary detection [31]. BPS, introduced as a substitute for BPA, has also demonstrated comparable endocrine-disrupting potential [31].

Parabens (Figure 1c) are *p*-hydroxybenzoic acid esters commonly used as preservatives in cosmetics, pharmaceuticals, and personal care products. Butylparaben (BP) and propylparaben (PP) are notable due to their relatively strong endocrine activity compared with shorter-chain parabens. In vivo, parabens undergo aromatic hydroxylation, yielding phenolic metabolites such as ethyl protocatechuate (EPA), which can be detected in urine and used to trace metabolic pathways [31].

Several substituted phenols were included because of their established relevance as phenolic EDCs (Figure 1c). 4-Hydroxybenzophenone (4-HBP), a metabolite of UV filters used in sunscreens, shows estrogenic activity and is detected in urine [32]. 3-Phenoxybenzoic acid (3-PBA) is the primary urinary metabolite of pyrethroid insecticides and serves as a widely used biomarker [33]. 4-Nitrophenol (PNP), a metabolite of organophosphate insecticides, and 2,4,5-trichlorophenol (TCP), a degradation product of chlorinated herbicides, both exhibit endocrine-disrupting properties [34]. Together, these twelve representative analytes provide a comprehensive basis for optimizing negative PSI–MS performance, which is expected to extend the applicability of the optimized method to a broader spectrum of EDCs and related biomarkers.

#### 2.1.2. Effects of Spraying Solvent and Additive on the Ionization Yield of Model Analytes

To identify the optimal spraying solvent and additive conditions for the 12 target analytes, their deprotonated-ion intensities were measured using selected reaction monitoring (SRM), as described in the Materials and Methods Section. First, we compared protic and aprotic solvents commonly used in PSI-MS. Specifically, MeOH, the most frequently applied protic solvent, and acetonitrile (ACN), a representative aprotic solvent, were tested for their ability to generate deprotonated ions of the analytes. As previously reported [6], the use of protic MeOH as the spraying solvent consistently produced higher signal intensities in negative PSI–MS for all 12 analytes compared with aprotic ACN (Figure S1). In addition, aqueous MeOH (MeOH/H_2_O, 9:1, *v*/*v*) generally yielded lower responses than pure MeOH. Based on these results, MeOH was selected as the base solvent for subsequent additive optimization.

Four additives previously reported to enhance negative ion formation in ESI-MS and PSI-MS were then evaluated: acetic acid [14], AmOH [13], AmF [12], and CTC [16,17]. In the initial screening, acetic acid did not provide a marked improvement relative to the other additives and was excluded from further investigation. The remaining three additives were examined in more detail, including potential synergistic effects between AmOH or AmF, both expected to promote deprotonation due to their basicity, and CTC, which can act as an electron scavenger by producing chlorine atoms.

**Figure 2:**
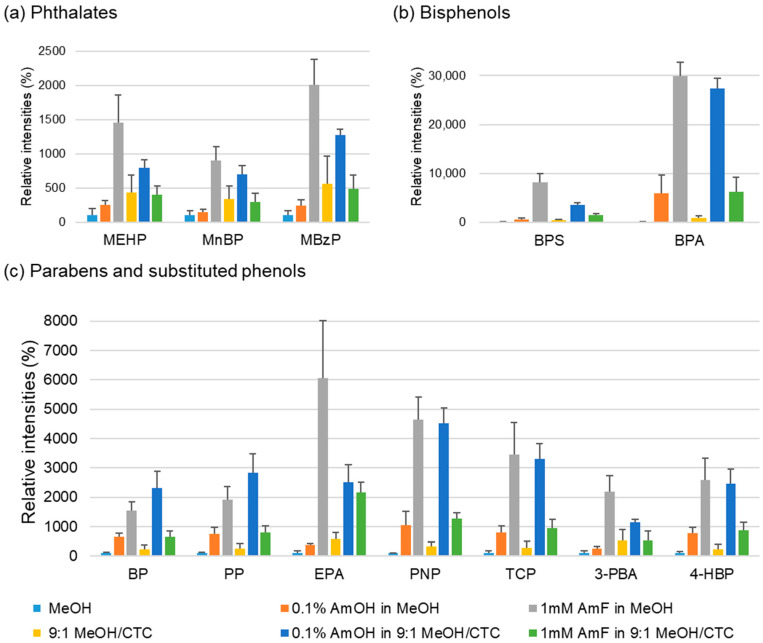
Mean deprotonated-ion intensities of 12 EDC analytes obtained by negative PSI-MS under six spraying solvent conditions: pure MeOH (sky blue), 0.1% AmOH in MeOH (orange), 1 mM AmF in MeOH (grey), 9:1 MeOH/CTC (yellow), 0.1% AmOH in 9:1 MeOH/CTC (blue), and 1 mM AmF in 9:1 MeOH/CTC (green). Bars represent average intensities from three replicate analyses, and error bars indicate %RSD. Intensities are normalized to those obtained with neat MeOH, which was set to 100%.

Accordingly, six PSI-MS spraying solvent conditions were compared for their ability to generate [M–H]^−^ ions from the 12 analytes: pure solvent (1) MeOH; single-additive systems, (2) AmOH, (3) AmF, and (4) CTC; and dual-additive systems, (5) AmOH + CTC and (6) AmF + CTC. For each additive, spraying solvents were prepared at three different concentrations with reference to ranges commonly applied in previous ESI-MS and PSI-MS studies. Optimal concentration of each additive was determined from the signal responses of the 12 analytes (Figures S2–S4). The deprotonated ion yields of the 12 target analytes, expressed as relative intensities under six spraying solvent conditions with the optimized additive concentrations, are presented in Figure 2.

The bar graph in Figure 2 presents the mean deprotonated-ion intensities of 12 EDC-related analytes measured under six selected spraying solvent conditions, with %RSD from three replicate analyses shown as error bars. Overall, MeOH solvents containing additives consistently produced higher signal intensities (1.4 to 300-fold enhancement) than pure MeOH, demonstrating that the incorporation of suitable additives effectively enhances negative-ion formation in PSI-MS.

Among the single-additive systems—AmOH (orange bar), AmF (grey bar), and CTC (yellow bar)—1 mM AmF in MeOH provided the highest average intensity across all 12 analytes, with the most pronounced relative improvement observed for bisphenols. The dual-additive system comprising 0.1% AmOH in 9:1 MeOH/CTC (blue bar) also showed strong performance. Although the mean intensities under this condition were generally slightly lower than those obtained with 1 mM AmF in MeOH, the enhancement relative to neat MeOH was substantial for all analytes. Notably, reproducibility improved with the AmOH/CTC system (mean %RSD across 12 analytes: 16%) compared to the single AmF additive (mean %RSD: 23%), suggesting that this condition stabilizes ion generation across replicate measurements.

Taken together, these results identify 1 mM AmF in MeOH and 0.1% AmOH in 9:1 MeOH/CTC as the most effective solvent systems for enhancing negative PSI-MS of the 12 EDC-related compounds. While AmF in MeOH generally yielded the strongest responses, the AmOH/MeOH–CTC mixture provided comparably high signals with reduced variability. Accordingly, both solvent systems were selected for subsequent validation of analytical performance. The enhanced signal intensity and stability observed under the optimized solvent conditions can be explained by two distinct mechanisms: the chemical effect of basic additives and the physical influence of the halogenated co-solvent. AmF and AmOH create a mildly basic microenvironment that facilitates deprotonation of hydroxyl and carboxyl groups, promoting the formation of stable [M–H]^−^ ions in both the solution and gas phases [10,11,12,13,14,15]. Meanwhile, CTC acts as an electron scavenger that captures free electrons generated near the paper tip, thereby suppressing corona discharge and stabilizing the spray plume [6,16,17]. The combined effect of enhanced deprotonation and reduced discharge instability results in stronger and more stable negative-ion signals.

#### 2.1.3. Initial Quantitative Evaluation of Two PSI Spraying Solvent Systems: Linearity and LOQ

To evaluate the basic quantitative performance of negative PSI–MS using the two spraying solvent systems selected from the intensity comparison of deprotonated ions of 12 EDC analytes (i.e., 1 mM AmF in MeOH and 0.1% AmOH in 9:1 MeOH/CTC), calibration curves were constructed and LOQs were determined with mixed standard solutions prepared at different concentrations. However, direct quantitation based on the absolute intensities of deprotonated ions exhibited relatively high variability (~20%), limiting reproducibility. To address this issue, all mixed standard solutions were prepared to include a fixed concentration (200 ppb) of internal standards (ISs). Three ISs were employed in this study: MEHP-d4 for phthalate analytes (Figure 1a), BPA-d16 for bisphenol analytes (Figure 1b), and BP-d4 for paraben and substituted phenol analytes (Figure 1c). Calibration curves and LOQ values were therefore derived from analyte-to-IS intensity ratios rather than absolute ion intensities.

Table 1 summarizes the calibration ranges, correlation coefficients (*R*^2^), and LOQs (ppb) obtained for the 12 target analytes using the two selected PSI spraying solvent systems. The corresponding calibration curves for each analyte are shown in Figures S5 and S6. All calibration curves were prepared up to 1000 ppb to provide an initial assessment of quantitative performance and to probe the limits of linearity. While most analytes maintained good linearity across this range (Table 1), slight upward deviation was observed at the highest point (1000 ppb) for specific compounds, including bisphenols with both spraying solvents, 3-PBA with AmF in MeOH, and EPA with AmOH in MeOH/CTC (Figures S5 and S6). These results indicate that although the PSI-MS with both spraying solvents demonstrates broad linearity, the validated linear range for reliable quantitation is generally confined to 20–500 ppb for certain analytes.

Across both spraying solvents, the LOQs for nearly all analytes fell within the sub-10 to 20 ppb range, demonstrating satisfactory sensitivity of the PSI-MS method. With 1 mM AmF in MeOH, several compounds such as BP, PP, EPA, and TCP achieved single-digit LOQs (<10 ppb), while most others remained well below 20 ppb. Comparable performance was observed with 0.1% AmOH in 9:1 MeOH/CTC, where the majority of analytes also yielded LOQs between 6 and 20 ppb. These results indicate that both solvent systems enable reliable low-ppb detection across diverse analyte classes, with only minor variations between compounds. In addition, the average percent bias of the low calibration standard (20 ppb), obtained from the back-calculated concentrations, is summarized in Table S1. Although the calibration curves were not fully optimized, all analytes except 3-PBA and 4-HBP showed biases within the acceptable range of ±20%, indicating satisfactory quantitative performance.

### 2.2. Application to Quantitative Analysis of Parabens in Cosmetics

The optimized negative PSI-MS method with the two selected spraying solvent systems was further evaluated for its applicability to real cosmetic matrices by targeting parabens as representative analytes. Parabens were considered suitable candidates since both solvent systems had already demonstrated comparable and satisfactory quantitative performance with standard solutions, as evidenced by their consistent linearity and low LOQs (Table 1). Moreover, a previous study has reported the feasibility of paraben analysis using MeOH as a PSI-MS spraying solvent [25], which provides additional support for their selection. While BP and PP were used as representative analytes during the optimization stage, methylparaben (MP) and ethylparaben (EP) were additionally included in the application study because regulatory authorities such as the European Union and South Korea impose concentration limits on all four parabens. According to these regulations, MP and EP are permitted up to 0.4% each, whereas PP and BP are restricted to 0.14%, with a maximum combined limit of 0.8% [35]. Although this study employed SRM-based quantitation monitoring four paraben-specific precursor/product ion transitions, the full mass spectrum of the hand cream sample fortified with four parabens and the tandem mass spectrum of the ion at *m/z* 179 corresponding to [PP–H]^−^ have been additionally provided as reference data in Figure S7.

**Figure 3:**
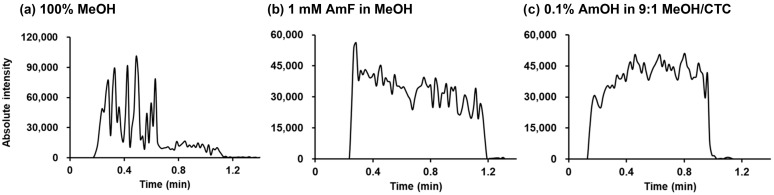
EICs of PP spiked into hand cream at 100 ppm (equivalent to 200 ppb in the solution after sample preparation), acquired by negative PSI-MS using different spraying solvents:.

For the four paraben analytes, excellent linearity (*R*^2^ > 0.998) was obtained with both selected spraying solvents across the concentration range of 20–500 ppb, as shown in Figures S8 and S9. Figure 3 presents the extracted ion chronograms (EICs) of PP fortified into the hand cream matrix, acquired by negative PSI-MS using pure MeOH and the two selected spraying solvents. As clearly shown, the generation of negative ions was markedly more stable with the optimized solvent systems compared to pure MeOH, demonstrating their superior performance in complex cosmetic matrices. Building on these results, we subsequently evaluated the analytical performance of the method in cosmetic product matrices.

To further assess the method performance, parabens were fortified into a blank (paraben-free) hand cream matrix, and LOQs were evaluated. In parallel, LOQs were also determined for the same fortified samples using LC-MS/MS, a widely adopted sensitive method for paraben analysis [36,37,38], and the results are summarized in Table 2. For PSI-MS, all the LOQs in solution after sample preparation were below 15 ppb (μg/L). When converted to matrix-based concentrations, the LOQs in the sample were consistently below 1 mg/kg. Between the two PSI solvent systems, 0.1% AmOH in 9:1 MeOH/CTC yielded slightly lower LOQs than 1 mM AmF in MeOH. Overall, the LOQs obtained by PSI-MS/MS were comparable to those obtained via LC-MS/MS for all paraben species. Moreover, when compared with the previous study employing pure MeOH as the spraying solvent [25], the use of 0.1% AmOH in 9:1 MeOH/CTC or 1 mM AmF in MeOH improved LOQs by nearly two orders of magnitude. This enhancement in sensitivity is attributed to both the increased absolute yield of negative ions (Figure 2c) and the improved signal stability (Figure 3).

In addition, the accuracy (%recovery) and intra- and inter-day precision (%RSD) of the developed PSI-MS method were further evaluated using paraben-fortified hand cream samples, and the results are presented in Table 3. For both PSI solvent systems, accuracy values ranged from 93% to 110%, demonstrating acceptable recovery. Intra- and inter-day precision were also below 10% RSD, with overall slightly better reproducibility observed when 0.1% AmOH in 9:1 MeOH/CTC was used as the spraying solvent. In addition to hand cream, accuracy and intra-day precision were also assessed for other cosmetic products, including toner, mist, and essence, with the results summarized in Table S1. These products also exhibited generally acceptable accuracy and precision; however, precision for MP was relatively lower, with recoveries ranging from 72% to 84%. This reduced performance is likely attributable to elevated background signals corresponding to the SRM transition of MP observed even in blank matrices. Consequently, further investigation into matrix effects, particularly potential interferences affecting MP quantitation, is warranted.

Taken together, these results demonstrate that the optimized PSI-MS conditions enable reliable quantitation of parabens in cosmetic products, with the 0.1% AmOH in 9:1 MeOH/CTC spraying solvent providing marginally superior overall performance compared with 1 mM AmF in MeOH.

## 3. Materials and Methods

### 3.1. Materials

BPA, BPS, MP, EP, PP, BP, EPA, 4-HBP, 3-PBA, MBzP, MEHP, MnBP, PNP, and TCP were purchased from Sigma-Aldrich (St. Louis, MO, USA). Reagents including AmOH (28–30% *w*/*w*), AmF, and CTC were also obtained from Sigma-Aldrich. LC-MS grade acetic acid was obtained from Tokyo Chemical Industry (Tokyo, Japan). HPLC-grade MeOH was purchased from Tedia (Fairfield, OH, USA), while HPLC-grade H_2_O was supplied by Samchun Pure Chemical (Pyeongtaek, Republic of Korea). Deuterium-labeled ISs, including BP-d4, MEHP-d4, and BPA-d16, were purchased from Toronto Research Chemicals (Toronto, ON, Canada). Whatman Grade 1 filter paper (0.18 mm thickness) was obtained from Whatman International Ltd. (Maidstone, UK), and 0.45 μm hydrophobic polytetrafluoroethylene (PTFE-D) syringe filters were purchased from Hyundai Micro (Anseong, Republic of Korea). Various paraben-free cosmetic products (hand cream, toner, mist, and essence) were procured from local markets and online stores.

### 3.2. Preparation of Standards for the Evaluation of Negative PSI-MS Performance

To optimize the performance of negative PSI-MS, stock solutions of twelve selected compounds (Figure 1) and ISs were prepared at a concentration of 1000 ppm in MeOH. For the experiments evaluating the absolute signal intensities of twelve analytes with different spraying solvents, a mixed standard solution was prepared by diluting each analyte in MeOH to yield a final concentration of 1 ppm for each compound. For the assessment of linearity and LOQ, calibration standard solutions were prepared in which the concentrations of the twelve target analytes ranged from 10 to 1000 ppb, while the concentrations of the ISs (BP-d4, MEHP-d4, and BPA-d16) were fixed at 200 ppb.

### 3.3. Standards and Sample Preparation for the Analysis of Parabens in Cosmetics

**Figure 4:**
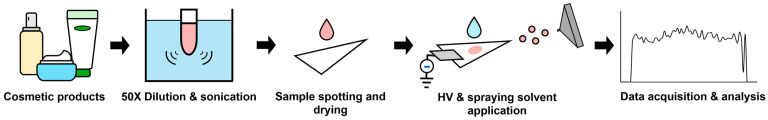
Workflow illustrating the analytical procedure for paraben determination in cosmetic products.

The analytical procedure for the determination of parabens in cosmetic products is illustrated in Figure 4, and a photograph of the PSI-MS platform is presented in Figure S10. Stock solutions of parabens (MP, EP, PP, and BP) and the internal standard (BP-d4) were prepared in MeOH at a concentration of 1000 ppm. For calibration curve construction, working standard solutions were prepared to yield paraben analyte concentrations ranging from 20 to 500 ppb, with the IS (BP-d4) fixed at 200 ppb.

For cosmetic product analysis, 0.1 g of sample was mixed with 4.9 g of MeOH and sonicated for 20 min. The resulting solution was used directly for PSI-MS analysis, whereas an additional filtration step using a PTFE-D syringe filter was performed prior to LC-MS/MS analysis. To evaluate LOQ, accuracy, and precision, paraben standards and IS were spiked into a paraben-free cosmetic sample to obtain final concentrations of 20 ppb for the paraben analytes and 200 ppb for the IS in analysis-ready solutions.

### 3.4. PSI-MS/MS

The PSI source was constructed in-house and interfaced with a TSQ Quantiva triple quadrupole mass spectrometer (Thermo Scientific Inc., San Jose, CA, USA). A triangular paper tip prepared from Whatman grade 1 filter paper (base: 5 mm, height: 10 mm) was secured to the high-voltage power supply via a clip. In PSI-MS/MS analysis, a 2 μL aliquot of the sample solution was deposited onto the central region of the paper tip. Following drying, the tip was mounted at an angle of approximately 30° and aligned 5 mm from the mass spectrometer inlet. Subsequently, a spray voltage of −3.0 kV was applied, and 10 μL of spraying solvent was dispensed onto the paper tip. As detailed in the Results and Discussion Section, six different spraying solvents were evaluated. Although minor variations were observed depending on the spraying solvent, under these conditions, the signal persisted for approximately 0.8–1.2 min. The mass spectrometer ion transfer capillary was operated at a temperature of 200 °C with an applied voltage of −35 V.

MS/MS data were acquired in SRM mode with unit mass resolution for both Q1 and Q3 (0.7 FWHM). The transition parameters, including collision energy (CE) and the *m*/*z* values of the precursor, quantifying, and qualifying ions for each analyte, are summarized in Table 4. In addition, the experimentally recorded SRM transitions for representative analytes are shown in Figure S11. For preparing calibration curves and determining concentrations, analyte-to-IS ratios were calculated. Specifically, MEHP-d4 was used as the internal standard for phthalates (Figure 1a), BPA-d16 for bisphenols (Figure 1b), and BP-d4 for parabens and substituted phenols (Figure 1c). A deviation within ±20% between the qualifier-to-quantifier ion ratios of the calibration standard and those of the sample was considered acceptable for compound confirmation.

### 3.5. LC-MS/MS

Reversed-phase LC separations of parabens were performed on a Nexera X2 LC-30AD system (Shimadzu, Kyoto, Japan) equipped with a Phenomenex Kinetex C18 column (100 × 2.1 mm, 2.6 μm; Phenomenex, Inc., Torrance, CA, USA). The mobile phases consisted of (A) 0.1% acetic acid in water and (B) 0.1% acetic acid in MeOH. Gradient elution was carried out by increasing the proportion of phase B from 45% to 60% over 0–4 min, followed by a linear increase to 90% over the next 2 min. The total run time, including re-equilibration, was 11 min. The flow rate was maintained at 400 μL/min, and the injection volume was 4 μL. The LC system was coupled to a TSQ Quantiva triple quadrupole mass spectrometer via a heated electrospray ionization (Thermo) source. The spray voltage was set to −3.5 kV, with vaporizer and ion transfer capillary temperatures of 250 °C and 275 °C, respectively. The capillary voltage was set to −35 V. Sheath gas and auxiliary gas pressures were adjusted to 45 and 10 arbitrary units, respectively. MS/MS data for parabens were acquired in SRM mode under the same conditions used for the PSI-MS/MS experiments (see Table 4).

## 4. Conclusions

This work demonstrates that careful solvent and additive optimization significantly improves the stability and sensitivity of negative PSI-MS. Using model endocrine-disrupting analytes, two optimized solvent systems were established, enabling low-ppb quantitation with strong reproducibility. Application of the method to cosmetic matrices confirmed reliable determination of parabens with accuracy and precision comparable to LC-MS/MS. These findings establish negative PSI-MS as a practical platform for paraben analysis in cosmetics. Future studies will extend this approach toward the detection of EDCs and exposure biomarkers not only in consumer products but also in human biological samples, supporting rapid ambient screening of chemicals relevant to public health.

## Figures and Tables

**Table 1:** Summary of calibration ranges, *R*^2^ values, and LOQs for selected analytes analyzed via negative PSI-MS with two spraying solvents.

Analytes	Spraying Solvents					
	1 mM AmF in MeOH			0.1% AmOH in 9:1 MeOH/CTC		
	Range (ppb)	*R* ^2^	LOQ (ppb) *	Range (ppb)	*R* ^2^	LOQ (ppb) *
MEHP	20–1000	0.9976	10.9	20–1000	0.9988	10.5
MnBP	20–1000	0.9980	14.7	20–1000	0.9986	11.3
MBzP	20–1000	0.9902	21.0	20–1000	0.9860	18.6
BPS	20–1000	0.9778	10.9	20–1000	0.9908	18.8
BPA	20–1000	0.9756	18.7	20–1000	0.9876	20.0
BP	10–1000	0.9997	6.6	10–1000	0.9975	6.7
PP	10–1000	0.9985	7.4	10–1000	0.9978	8.1
EPA	10–1000	0.9983	4.1	10–1000	0.9818	7.2
PNP	10–1000	0.9927	8.1	10–1000	0.9924	6.3
TCP	10–1000	0.9980	7.6	10–1000	0.9980	9.3
3-PBA	10–1000	0.9638	4.0	20–1000	0.9976	16.3
4-HBP	10–1000	0.9983	6.9	10–1000	0.9996	7.6

* LOQ values were calculated as 10σ/m, where σ represents the standard deviation of replicate measurements (*n* = 7) at low concentrations and m is the slope of the calibration curve.

**Table 2:** LOQs in solution (ppb) and in sample (mg/kg) for paraben analytes in hand cream, determined via negative PSI-MS/MS with two spraying solvent systems and via LC-MS/MS.

Analytes	LOQ in Solution (ppb) ^1^			LOQ in Sample (mg/kg) ^2^		
	PSI-MS/MS (AmF)	PSI-MS/MS (AmOH/CTC)	LC-MS/MS	PSI-MS/MS (AmF)	PSI-MS/MS (AmOH/CTC)	LC-MS/MS
MP	12.6	8.4	5.8	0.7	0.4	0.3
EP	8.0	5.8	11.7	0.4	0.3	0.6
PP	6.8	4.3	5.0	0.4	0.2	0.3
BP	11.4	5.8	8.5	0.6	0.3	0.4

^1^ LOQs in solution were determined by analyzing paraben-fortified hand cream, yielding a final analyte concentration of 20 ppb in the solution obtained after sample preparation. LOQ values were calculated as 10 σ/m, where σ is the standard deviation of replicate measurements (*n* = 7) at 20 ppb and m is the slope of the calibration curve. ^2^ LOQs in the sample represent the LOQs in solution converted to mg per kg of cosmetic product prior to sample preparation.

**Table 3:** Accuracy (%recovery), intra-day (*n* = 3) and inter-day (*n* = 3) precision (%RSD) for paraben analytes in hand cream, determined via negative PSI-MS/MS with two spraying solvent systems and via LC-MS/MS. Accuracy and precision were estimated by analyzing paraben-fortified hand cream, yielding a final analyte concentration of 20 ppb in the solution obtained after sample preparation.

Analytes	Accuracy (%)			Intra-/Inter-day Precision (%RSD)		
	PSI-MS/MS (AmF)	PSI-MS/MS (AmOH/CTC)	LC-MS/MS	PSI-MS/MS (AmF)	PSI-MS/MS (AmOH/CTC)	LC-MS/MS ^1^
MP	93	100	77	4.2/6.0	3.7/5.1	4.0/-
EP	109	110	90	2.5/5.4	2.8/4.4	7.0/-
PP	109	106	110	3.4/3.9	2.6/4.2	7.0/-
BP	104	102	94	4.0/6.6	1.1/3.7	5.9/-

^1^ For LC-MS/MS, only intra-day precision was estimated.

**Table 4:** SRM condition used in this study.

Analytes and IS	Precursor Ion	Quantifying Ion	CE (eV)	Qualifying Ion	CE (eV)
MP	151	92	26	136	17
EP	165	92	24	136	16
BP	193	92	25	136	18
PP	179	92	23	136	16
EPA	181	108	21	153	29
PNP	138	108	22	137	25
TCP	195	159	20	123	28
3-PBA	213	93	23	169	13
4-HBP	197	92	36	161	24
MEHP	277	134	18	127	18
MnBP	221	177	11	77	18
MBzP	255	77	21	183	13
BPS	249	108	29	92	42
BPA	227	212	18	133	33
BP-d4	197	96	26		
MEHP-d4	281	138	19		
BPA-d16	241	223	20		

## Data Availability

The original contributions presented in this study are included in the article/Supplementary Material. Further inquiries can be directed to the corresponding author(s).

## Supplementary Materials

The following supporting information can be downloaded at https://www.mdpi.com/article/10.3390/molecules30224356/s1. Quantitative performance measures of PSI-MS/MS methods against cosmetic product matrices, bar plots for PSI spraying solvent and additive optimization, and calibration curves. Figure S1: Comparison of relative intensities obtained using MeOH, MeOH/H_2_O (90/10, *v*/*v*), and ACN as spraying solvents for the analysis of model compounds by negative PSI-MS; Figure S2: Relative intensities of deprotonated ions from 12 model compounds as a function of AmOH concentration; Figure S3: Relative intensities of deprotonated ions from 12 model compounds as a function of AmF concentration; Figure S4: Relative intensities of deprotonated ions from 12 model compounds as a function of CTC concentration; Figure S5: Calibration curves for 12 EDC-related analytes obtained by negative PSI-MS using 1 mM AmF in MeOH as the spraying solvent; Figure S6: Calibration curves for 12 EDC-related analytes obtained by negative PSI-MS using 0.1% AmOH in 9:1 MeOH/CTC as the spraying solvent; Figure S7: Full mass spectrum of the hand cream sample fortified with four parabens and full tandem mass spectrum of the ion at *m*/*z* 179 corresponding to [PP – H]^−^; Figure S8: Calibration curves for 4 paraben analytes obtained by negative PSI-MS using 1 mM AmF in MeOH as the spraying solvent; Figure S9: Calibration curves for 4 paraben analytes obtained by negative PSI-MS using 0.1% AmOH in 9:1 MeOH/CTC as the spraying solvent; Figure S10: A photo of the PSI-MS platform used in this study; Figure S11: Experimentally recorded SRM transitions for representative analytes; Table S1: Average percent bias of the low calibration standard (20 ppb) based on back-calculated concentrations from the calibration curve (*n* = 3); Table S2: Accuracy (% recovery) and intra-day (*n* = 3) precision (%RSD) for paraben analytes in (a) toner, (b) mist, and (c) essence determined by negative PSI-MS/MS.

## Abbreviations

The following abbreviations are used in this manuscript:

ACN	Acetonitrile
AmF	Ammonium fluoride
AmOH	Ammonium hydroxide
BP	Butylparaben
BPA	Bisphenol A
BPS	Bisphenol S
CE	Collision Energy
CTC	Carbon tetrachloride
EDC	Endocrine-disrupting chemicals
EIC	Extracted ion chronogram
EP	Ethylparaben
EPA	Ethyl protocatechuate
ESI	Electrospray ionization
4-HBP	4-Hydroxybenzophenone
IS	Internal standard
LC	Liquid chromatography
LOQ	Limit of quantitation
MBzP	Monobenzyl phthalate
MEHP	Mono-2-ethylhexyl phthalate
MeOH	Methanol
MnBP	Mono-n-butyl phthalate
MP	Methylparaben
MS	Mass spectrometry
MS/MS	Tandem mass spectrometry
3-PBA	3-Phenoxybenzoic acid
4-PNP	4-Nitrophenol
PP	Propylparaben
PSI	Paper spray ionization
PTFE-D	Hydrophobic polytetrafluoroethylene
TCP	2,4,5-trichlorophenol
