# Supplementary material for: Negative Paper Spray Ionization Mass Spectrometry for the Determination of Endocrine-Disrupting Chemicals with Application to Paraben Analysis in Cosmetics
Source: Molecules. 2025 Nov 10;30(22):4356. doi: 10.3390/molecules30224356 (PMC12654611; doi:10.3390/molecules30224356)
Supplement: Supplementary file 1 [file molecules-30-04356-s001.zip › molecules-3920488-supplementary.pdf]

Supporting Information

Negative paper spray ionization mass spectrometry for the determination of endocrine disrupting chemicals with application to paraben analysis in cosmetics

Seonyoung Cho [1,†], Sarmila Shrestha Amatya [1,†], Hyerin Bahng [1], Eungyeong Lee [1], Yunsang Ko [1], and Sangwon Cha [1,*]

[1]Department of Chemistry, Dongguk University, Seoul 04620, Republic of Korea; chasw@dongguk.edu

† These authors contributed equally to this work.

*Correspondence: chasw@dongguk.edu; Tel.: +82-2-2260-8907 (S.C.)

Table S1. Average percent bias of the low calibration standard (20 ppb) based on back-calculated concentrations from the calibration curve (n = 3)

	Spraying solvents	
Analytes	1 mM AmF in MeOH	0.1% AmOH in 9:1 MeOH/CTC
MEHP	−9.1%	−12.2%
MnBP	−1.7%	−6.2%
MBzP	−4.0%	+3.6%
BPS	+8.5%	−1.7%
BPA	+14.7%	+16.1%
BP	−13.1%	−12.1%
PP	−7.3%	−7.6%
EPA	−14.9%	−2.5%
PNP	+15.7%	+14.1%
TCP	−4.2%	−2.4%
3-PBA	+30.2%	−20.4%
4-HBP	−21.6%	−19.7%

Table S2. Accuracy (% recovery), intra-day (n = 3) precision (%RSD) for paraben analytes in (a) toner, (b) mist, and (c) essence determined by (–) PSI-MS/MS with two spraying solvent systems and by (–) LC-MS/MS. Accuracy and precision were estimated by analyzing paraben-fortified cosmetic products, yielding a final analyte concentration of 20 ppb in the solution obtained after sample preparation.

(a) Toner

Analytes	Accuracy (%)			Intra-day Precision (%RSD)		
	PSI-MS/MS (AmF)	PSI-MS/MS (AmOH/CTC)	LC-MS/MS	PSI-MS/MS (AmF)	PSI-MS/MS (AmOH/CTC)	LC-MS/MS
MP	82	76	76	9.7	4.1	11.3
EP	101	89	90	6.3	1.7	6.5
PP	111	95	94	4.8	2.3	1.9
BP	109	102	94	4.1	2.4	4.6

(b) Mist

Analytes	Accuracy (%)			Intra-day Precision (%RSD)		
	PSI-MS/MS (AmF)	PSI-MS/MS (AmOH/CTC)	LC-MS/MS	PSI-MS/MS (AmF)	PSI-MS/MS (AmOH/CTC)	LC-MS/MS
MP	80	84	73	3.3	1.3	1.5
EP	103	97	85	5.9	1.6	4.7
PP	107	101	84	4.6	1.7	4.0
BP	102	104	80	2.9	2.1	2.3

(c) Essence

Analytes	Accuracy (%)			Intra-/Inter-day Precision (%RSD)		
	PSI-MS/MS (AmF)	PSI-MS/MS (AmOH/CTC)	LC-MS/MS	PSI-MS/MS (AmF)	PSI-MS/MS (AmOH/CTC)	LC-MS/MS
MP	72	74	77	1.1	6.4	2.6
EP	94	91	97	2.2	4.6	3.1
PP	105	99	113	3.3	2.1	2.7
BP	102	106	98	1.2	1.1	2.3

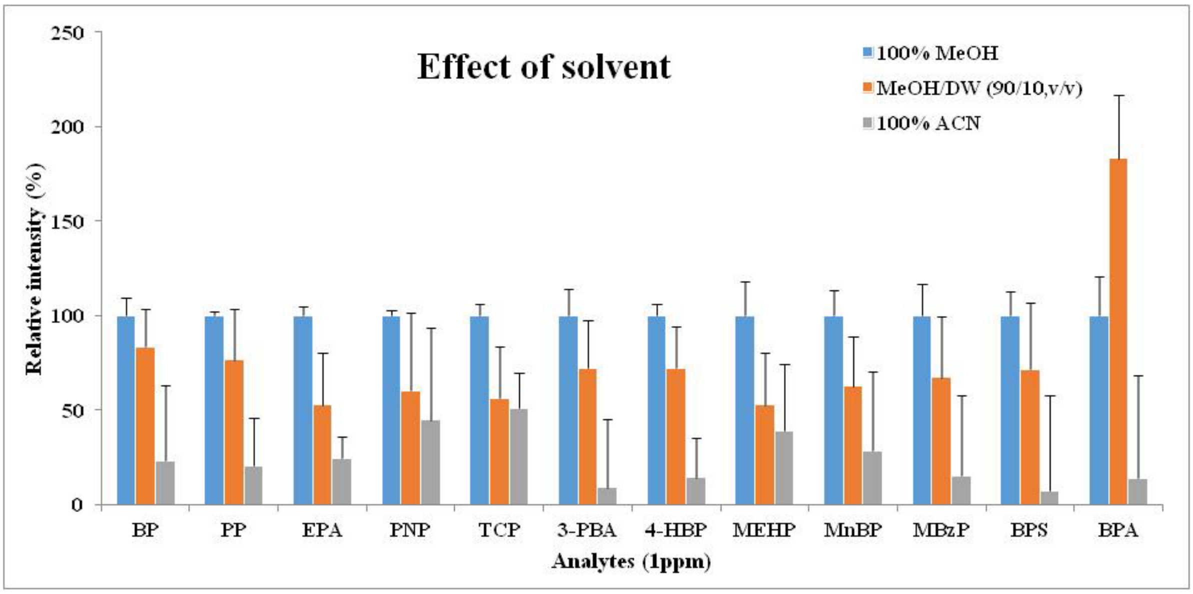

Figure S1. Comparison of relative intensities obtained using MeOH (blue), MeOH/H_2O (90/10, v/v) (orange), and ACN (grey) as spraying solvents for the analysis of model compounds by (−) PSI-MS. Intensities are expressed relative to those obtained with MeOH (set to 100%). Error bars represent %RSD from triplicate measurements.

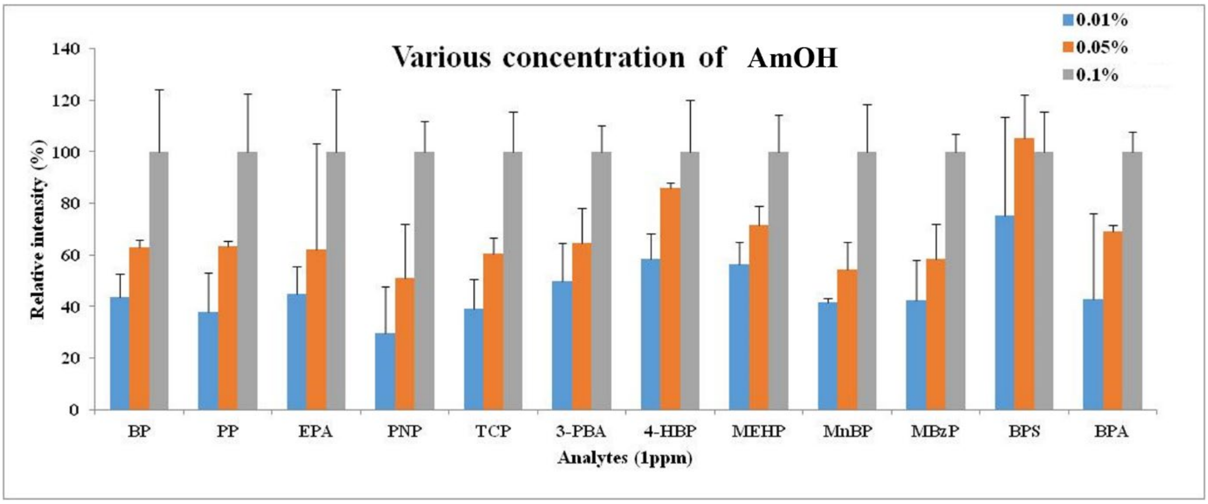

Figure S2. Relative intensities of deprotonated ions from 12 model compounds as a function of ammonium hydroxide (AmOH) concentration in MeOH (vol%): 0.01% (blue), 0.05% (orange), and 0.1% (grey). Intensities are normalized to those obtained with 0.1% AmOH in MeOH (set to 100%). Error bars represent %RSD from triplicate measurements. For most analytes, the highest responses were observed with 0.1% AmOH in MeOH.

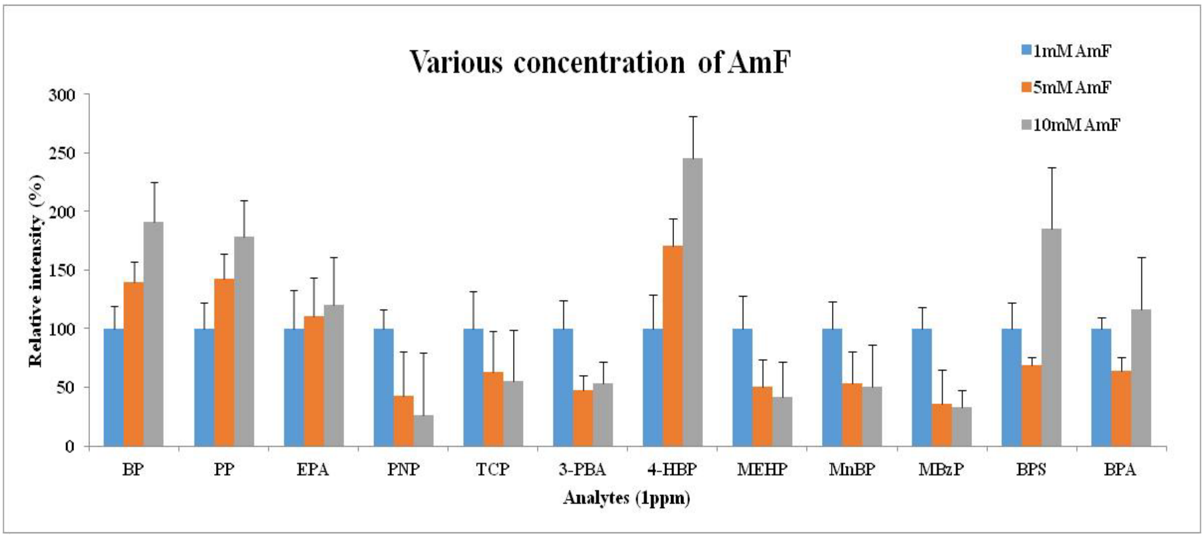

Figure S3. Relative intensities of deprotonated ions from 12 model compounds as a function of ammonium fluoride (AmF) concentration (mM) in MeOH: 1 mM (blue), 5 mM (orange), and 10 mM (grey). Intensities are normalized to those obtained with 1 mM AmF in MeOH (set to 100%). Error bars represent %RSD from triplicate measurements. Six of the twelve analytes showed the highest responses with 1 mM AmF, whereas the other six exhibited the highest responses with 10 mM AmF. Despite these mixed results, 1 mM AmF was selected for further investigation in this study due to its relatively lower variability in signal intensities.

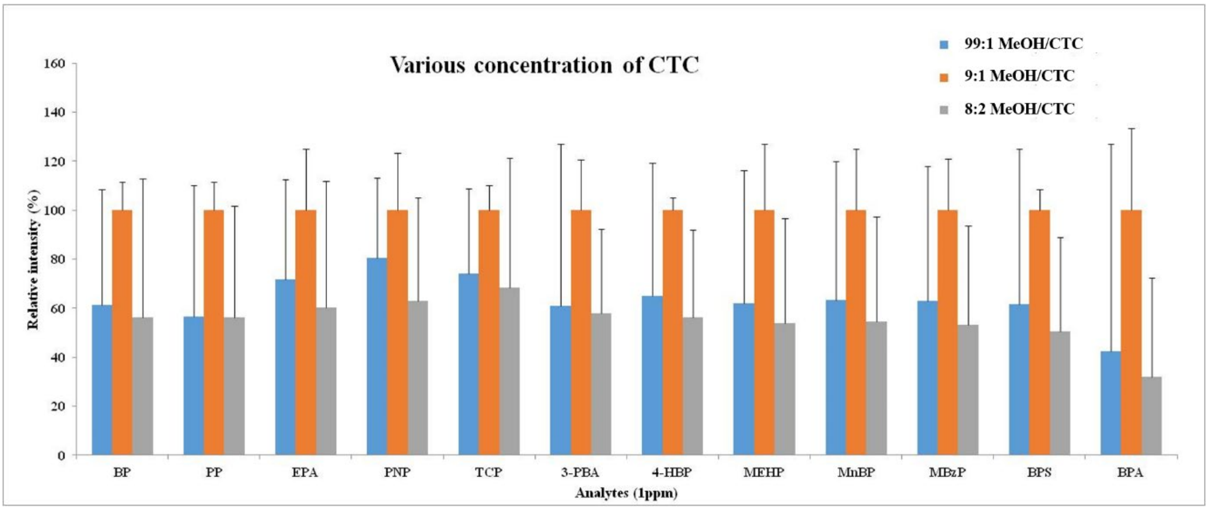

Figure S4. Relative intensities of deprotonated ions from 12 model compounds as a function of carbon tetrachloride (CTC) concentration in MeOH (v/v): 99:1 (blue), 9:1 (orange), and 8:2 (grey) MeOH/CTC. Intensities are normalized to those obtained with 9:1 MeOH/CTC (set to 100%). Error bars represent %RSD from triplicate measurements. The majority of analytes exhibited their highest responses with 9:1 MeOH/CTC.

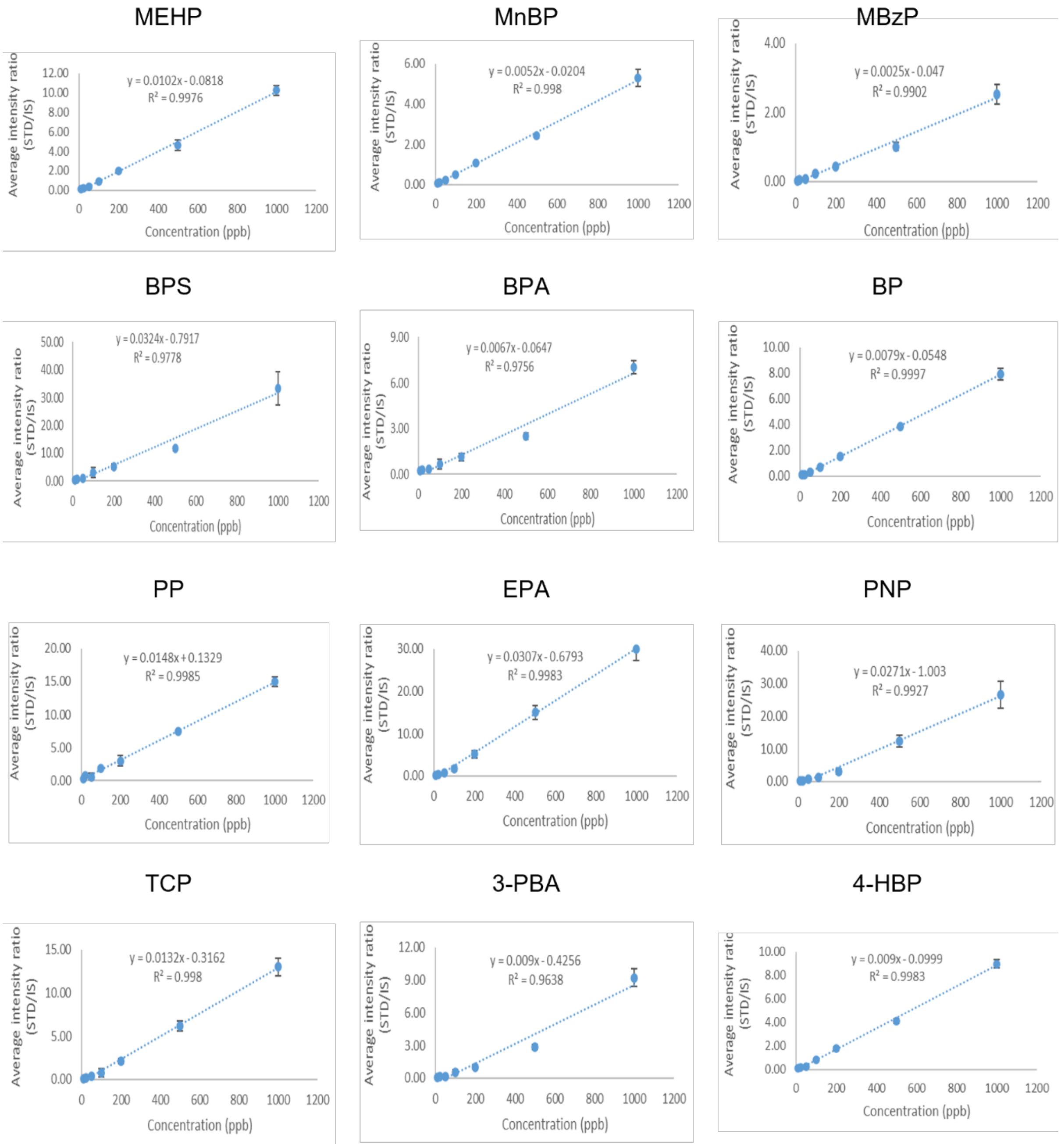

Figure S5. Calibration curves for 12 EDC-related analytes obtained by (–) PSI-MS using 1 mM AmF in MeOH as the spraying solvent.

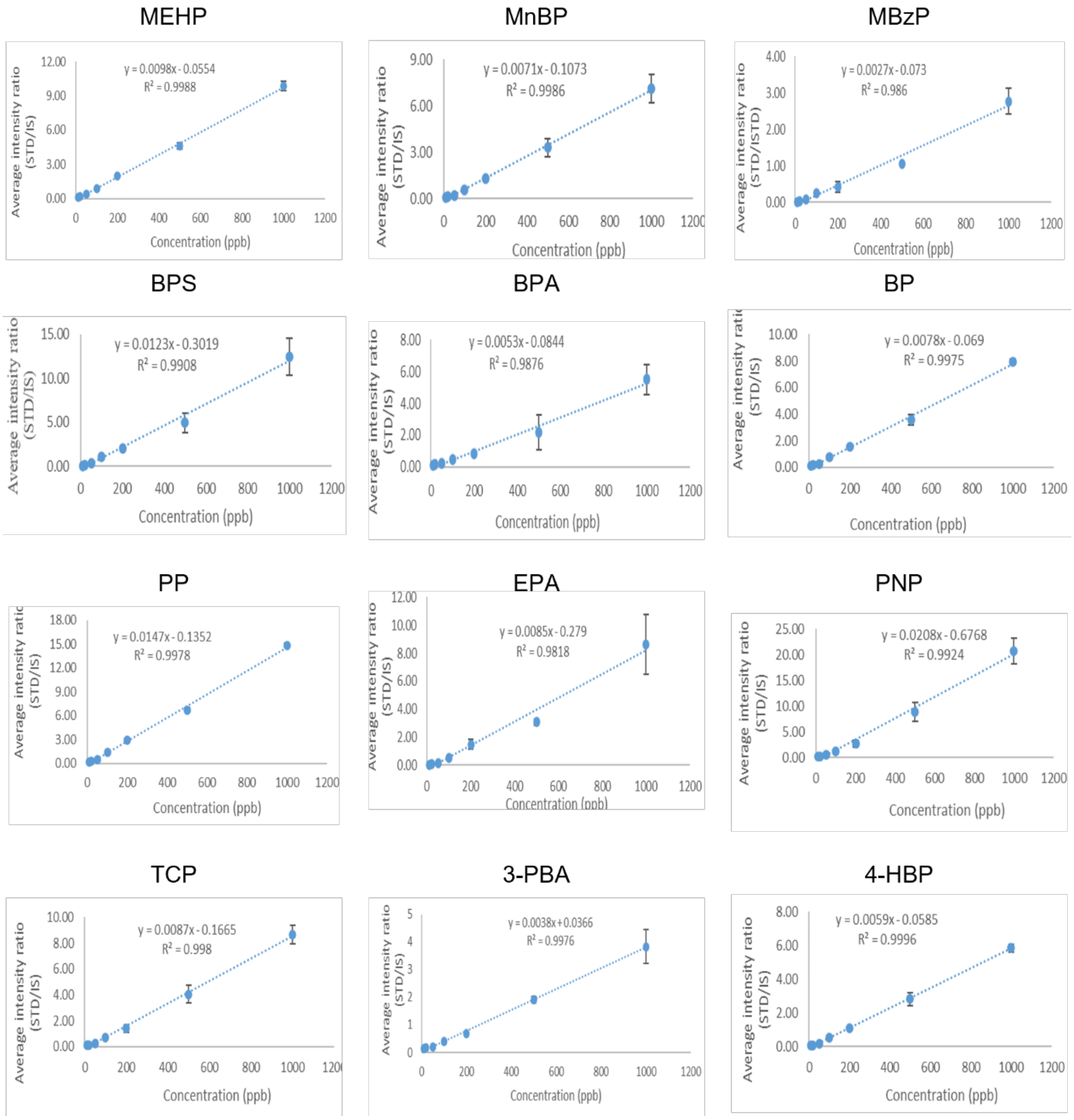

Figure S6. Calibration curves for 12 EDC-related analytes obtained by (–) PSI-MS using 0.1% AmOH in 9:1 MeOH/CTC as the spraying solvent.

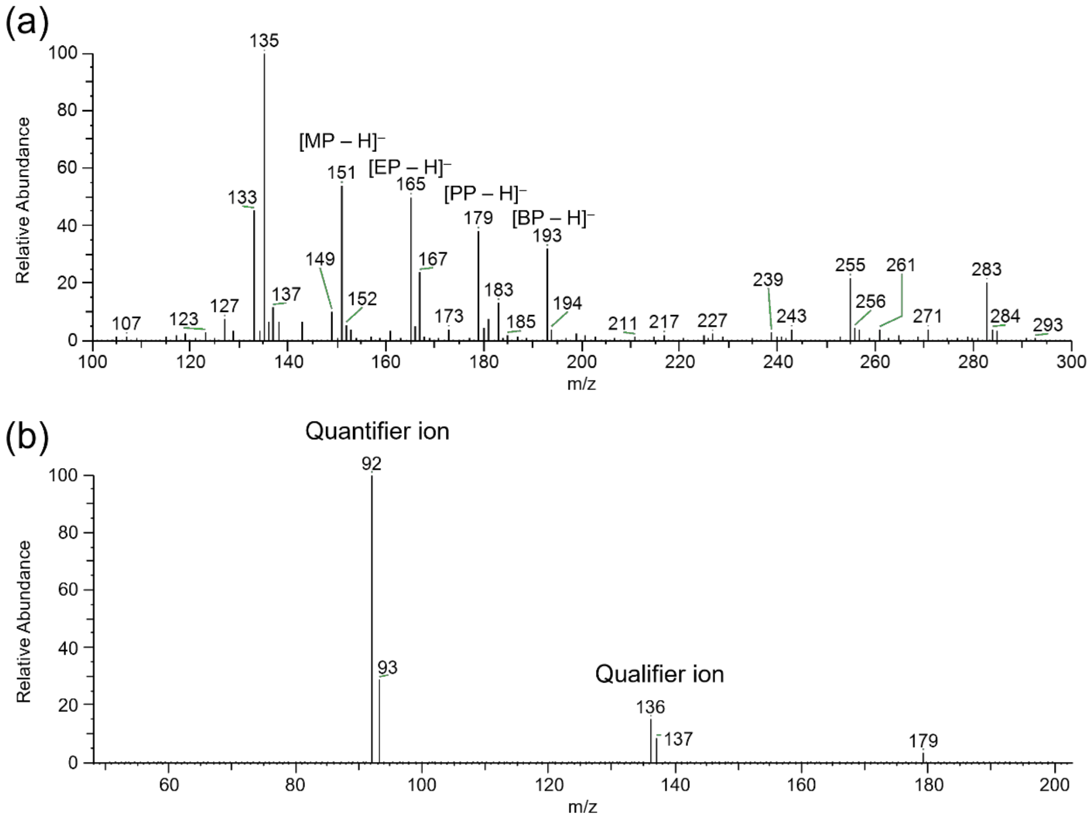

Figure S7. (a) Full mass spectrum of the hand cream sample fortified with four parabens and (b) full tandem mass spectrum of the ion at *m/z* 179 corresponding to [PP – H]$^-$

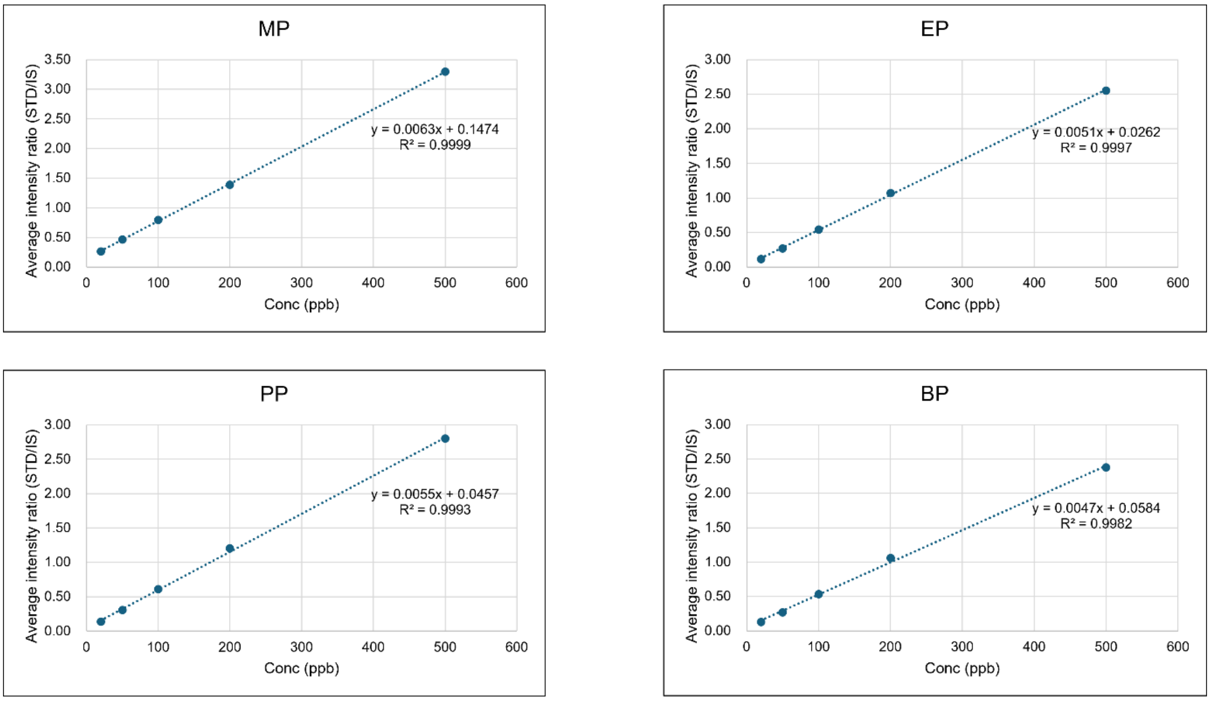

Figure S8. Calibration curves for 4 paraben analytes obtained by (–) PSI-MS using 1 mM AmF in MeOH as the spraying solvent.

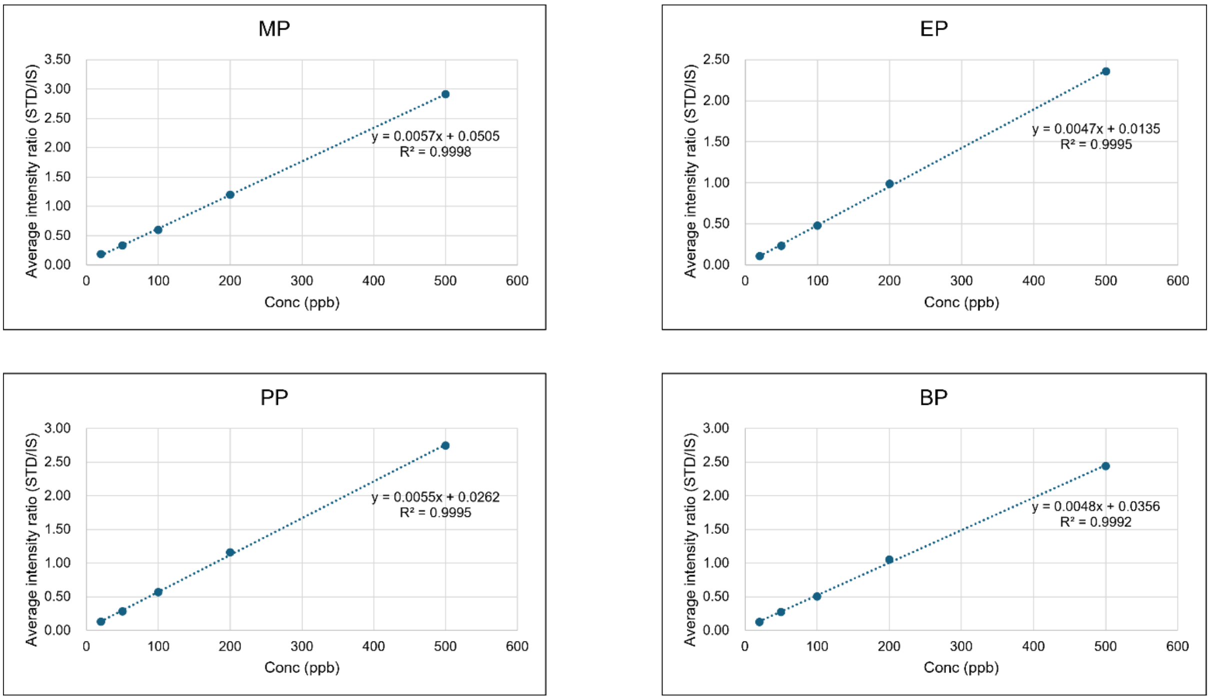

Figure S9. Calibration curves for 4 paraben analytes obtained by (–) PSI-MS using 0.1% AmOH in 9:1 MeOH/CTC as the spraying solvent.

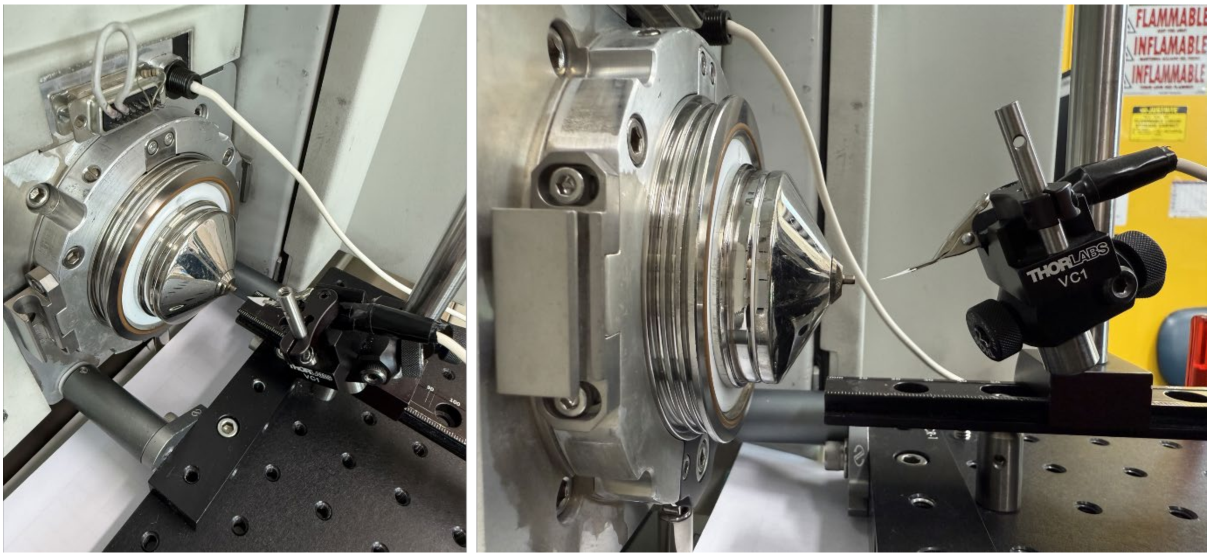

Figure S10. A photo of the paper spray ionization mass spectrometry (PSI-MS) platform used in this study.

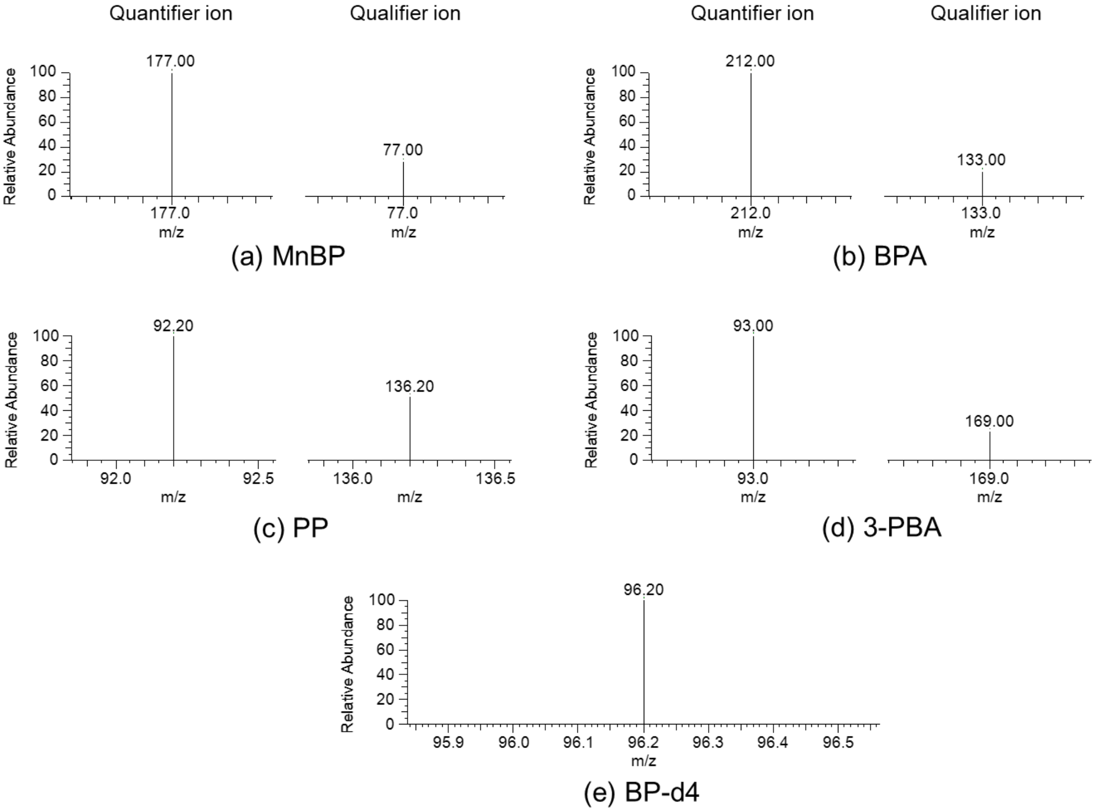

Figure S11. Experimentally recorded SRM transitions for representative analytes: (a) MnBP, (b) BPA, (c) PP, (d) 3-PBA, and (e) the internal standard BP-d4.
